# Determining gestational age for public health care users in Brazil: comparison of methods and algorithm creation

**DOI:** 10.1186/1756-0500-6-60

**Published:** 2013-02-13

**Authors:** Ana Paula Esteves Pereira, Marcos Augusto Bastos Dias, Maria Helena Bastos, Silvana granado Nogueira da Gama, Maria do Carmo Leal

**Affiliations:** 1 Escola Nacional de Saúde Pública Sergio Arouca, Fundação Oswaldo Cruz, Rio de Janeiro, Brasil; 2 Instituto Fernandes Figueira, Fundação Oswaldo Cruz, Rio de Janeiro, Brasil; 3 Pan-american Health Organization, World Health Organization, Brasília, Brasil

**Keywords:** Gestational age estimation, Prematurity rate, Last menstrual period, Ultrasound, Capurro method, Algorithm creation

## Abstract

**Background:**

A valid, accurate method for determining gestational age (GA) is crucial in classifying early and late prematurity, and it is a relevant issue in perinatology. This study aimed at assessing the validity of different measures for approximating GA, and it provides an insight into the development of algorithms that can be adopted in places with similar characteristics to Brazil. A follow-up study was carried out in two cities in southeast Brazil. Participants were interviewed in the first trimester of pregnancy and in the postpartum period, with a final sample of 1483 participants after exclusions. The distribution of GA estimates at birth using ultrasound (US) at 21–28 weeks, US at 29+ weeks, last menstrual period (LMP), and the Capurro method were compared with GA estimates at birth using the reference US (at 7–20 weeks of gestation). Kappa, sensitivity, and specificity tests were calculated for preterm (<37 weeks of gestation) and post-term (>=42 weeks) birth rates. The difference in days in the GA estimates between the reference US and the LMP and between the reference US and the Capurro method were evaluated in terms of maternal and infant characteristics, respectively.

**Results:**

For prematurity, US at 21–28 weeks had the highest sensitivity (0.84) and the Capurro method the highest specificity (0.97). For postmaturity, US at 21–28 weeks and the Capurro method had a very high sensitivity (0.98). All methods of GA estimation had a very low specificity (≤0.50) for postmaturity. GA estimates at birth with the algorithm and the reference US produced very similar results, with a preterm birth rate of 12.5%.

**Conclusions:**

In countries such as Brazil, where there is less accurate information about the LMP and lower coverage of early obstetric US examinations, we recommend the development of algorithms that enable the use of available information using methodological strategies to reduce the chance of errors with GA. Thus, this study calls into attention the care needed when comparing preterm birth rates of different localities if they are calculated using different methods.

## Background

To determine gestational age (GA), clinicians rely on various antenatal and postnatal indicators, such as the last menstrual period (LMP) and/or birth weight and first-trimester ultrasound (US). Dating GA based on the LMP is a simple, low-cost method [1]. The LMP is a universally available piece of self-reported information and is the method most used to estimate GA, particularly in developing countries; it is also the method recommended by the World Health Organization [2]. However, it has been recognized that this method of estimating GA is fallible under many circumstances, such as irregularity or individual variations in the length of the menstrual cycle, short birth spacing, preconception amenorrhea after oral contraceptive use, implantation bleeding, and recall biases by the mother [3-5]. In both developed and developing countries, reliance on LMP alone has shown a tendency to overestimate GA at the extremes of gestation owing to recall bias, thereby overestimating postmaturity and underestimating preterm births [4,6-11]. In developing countries, including Brazil, a large proportion of the population is of a low educational level—a well-known characteristic associated with poorer quality of LMP information [7,12]. In developed countries, most women have access to at least one early US during pregnancy, and it is common for clinicians to confirm menstrual dates using this information [1].

According to the National Institute for Health and Clinical Excellence (NICE) of the United Kingdom, an US check performed at 10 weeks to 13 weeks 6 days of gestation is considered the most accurate method for estimating GA because the variation in fetal growth rate is very low in this period [13]. In most developing countries, seeking help from health services in the diagnosis of pregnancy and initiating antenatal care tends to be delayed, which hinders conducting US checks in the early stages of pregnancy [14-16]. Moreover, in many countries, including Brazil, it is difficult to have immediate access to this type of examination [17].

Since the 1990s, several algorithms have been proposed, mainly in developed countries, aiming at calculating the GA using different available data sources. However, for countries with less favorable socioeconomic conditions, validation studies are still needed given the different characteristics of their populations in terms of access to social and health care services.

Therefore, we conducted the analysis presented here, which compares estimates of GA at birth using early US with such estimates obtained using later US examinations, LMP, the Capurro method, and anthropometric measures [18]. Our goal was to assess the validity of these measures for approximating GA among users of the public health sector from two municipalities of Rio de Janeiro State, Brazil. We also aimed to provide an insight into the development of algorithms to be adopted in places with similar characteristics.

## Methods

### Design and sample size

A prospective cohort study was conducted with pregnant women in two urban middle-sized cities (populations between 100,000 and 300,000) in the state of Rio de Janeiro, Brazil. Since the number of pregnant women available for analysis was based on the number available on the primary study (based on the prevalence of low birth weight), post-hoc sample size calculation was performed. Considering the prevalence of 10% of prematurity in Rio de Janeiro State, the postpartum sample (1483) had 90% power to detect differences of at least 5%.

### Study participants

The selection criteria included pregnant women in the first trimester of pregnancy living in one of the two cities who sought antenatal care in public health care units (state-funded) from December 2007 to November 2008. The antenatal care units selected included both primary and referral health centers and were identified through the Brazilian National Register of Health Establishments. All eligible women in these units were invited to participate and informed about the aims of this study. Participants gave their consent and were interviewed at three antenatal care units in city 1 and eight antenatal care units in city 2, which correspond to 90% of the public antenatal care coverage in both cities.

Different strategies were established in the study to reduce losses to follow-up, including particular attention in obtaining contact details of the participants. Furthermore, all women were requested to make a free call to one of the research workers to inform them when they were admitted to a maternity unit to give birth. The women were also asked to inform the researchers when changing addresses or telephone numbers.

**Figure 1:**
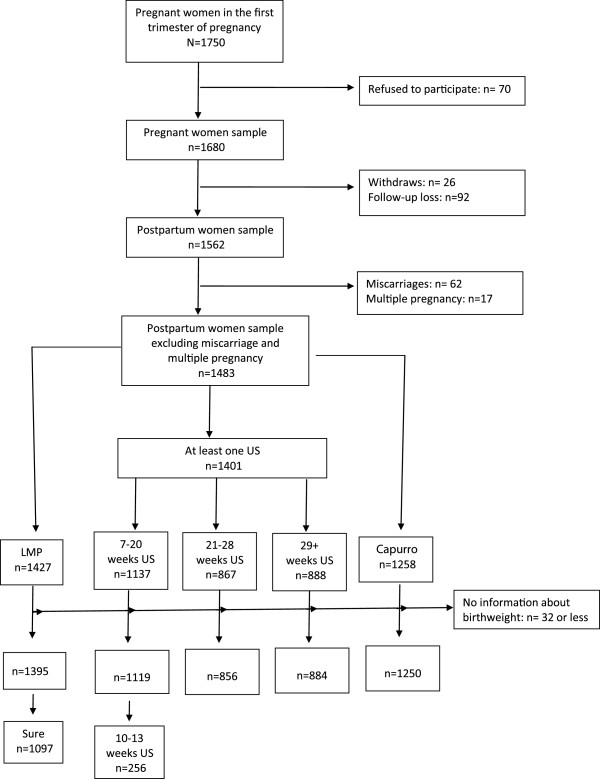
The flow chart of the sample.

Overall, 1750 pregnant women were invited to participate, and 1680 (96%) were enrolled and available for assessment. We excluded 118 women (7.5%) owing to withdrawal or loss to follow-up, 62 (3.7%) owing to miscarriage, and 30 (1%) owing to multiple pregnancies. This analysis includes 1483 singleton pregnancies with additional exclusions made when data were missing for each method of GA estimation used in the comparison (Figure 1).

This study is in compliance with the Helsinki Declaration and was approved by the Committee of Ethics and Research of the National School of Public Health—ENSP/FIOCRUZ (protocol no 158/06).

### Data collection

At the baseline, in a face-to-face interview, the women reported their demographic and socioeconomic characteristics, obstetric history, maternal pregestational anthropometric measures, and LMP. The women were asked, “What was the first day of your last menstrual period?” If necessary, they were prompted by being asked to think of an event that happened around the time of their last period (e.g., a holiday, vacation, or weekend) to facilitate recall. The mean GA at the first interview was 12.9 weeks (3–16 weeks). At this interview, data relating to US examinations were copied directly from the original results kept by the pregnant women.

The postpartum data collection took place at the health care units (80%) or at the women’s homes up to 30 days after hospital discharge (20%). Information concerning the mode of birth, sex of the neonate, neonatal birth weight and length, and GA estimates using the Capurro method (carried out by neonatologists according to the standard procedure of the health center and registered in the medical records) were collected from medical records when the mothers were interviewed during their hospital stay and from the birth certificate if they were interviewed after hospital discharge. The data for the birth certificate are transcribed from medical records and are of high quality in Rio de Janeiro State. In both cases, US data from previous examinations were copied directly from original results kept by the mothers.

In city 1, the majority of US examinations (90%) were performed by clinical sonographers at the public hospital where most of the women were interviewed. In city 2, the majority of US examinations (80%) were performed by a clinical sonographer from our research team.

#### GA, birth weight and weight-for-GA assessment

The GA at birth estimated from the LMP was calculated by subtracting the LMP from the date of birth [19]. For the US examinations, it was calculated by subtracting the date of the examination from the date of birth and adding the estimated clinical GA in days at the time of examination.

The US results were grouped into three gestational periods: the first, at 7–20 weeks of gestation; the second, at 21–28 weeks of gestation; and the third, at 29 and more weeks of gestation. As the reference method for estimating GA at birth, we took the earliest US result, performed at 7–20 weeks of gestation. According to NICE, the most accurate method is the US examination performed at 10 weeks to 13 weeks 6 days of gestation [13]. However, in the public health care sector in Brazil, a high proportion of women initiate antenatal care after this period. We opted to include US results of up to 20 weeks GA in the reference group since before 20 weeks of gestation, some authors consider US a more accurate method than the LMP [20-23]. US conducted at 21–28 weeks signifies the remainder of the second trimester and US conducted at 29 weeks and beyond refers to the third trimester of pregnancy, during which US examination is prone to larger errors. For the second- and third-gestational period US examinations, we selected the earliest one; for example, if a woman had one US at 22 weeks of gestation and another at 27, we used the information from the former.

The GA at birth estimated using the Capurro method was obtained from medical records as well as the birth weight, which was categorized as follows: <2500 g, 2500–3499 g, 3500–3999 g, and ≥4000 g. Estimating the GA using the Capurro method was carried out by neonatologists according to the standard procedure of the health center, and the results were registered in the medical records. It is based on an inspection of physical signs during the first 12 hours of life; the following five characteristics of the newborn are examined and placed on a maturation scale: skin texture, shape of the ear, size of the mammary nodule, formation of the nipple, and the folds of the sole of the foot.

The Z scores of birth weight for GA were calculated using the American curve of intrauterine growth divided by sex [24]. All newborns classified under −3 standard deviation (SD) or over +3 SD were grouped into two categories (≤ −3 SD outliers and ≥ +3 SD outliers) for graphical presentation purposes.

#### Statistical analysis

We compared the distribution of GA estimates at birth using the reference US with the estimates using US at 21–28 weeks, US at 29+ weeks, LMP, and the Capurro method. Prematurity (<37 weeks of gestation) and postmaturity (>=42 weeks of gestation) rates were compared using chi-square tests with a significance level of 0.05. For kappa, sensitivity, specificity, positive predictive value, and negative predictive value were compared between women for whom the GA was estimated by the reference US and by the other method. All comparisons were performed including and excluding outliers of Z scores for birth weight for GA. The agreement rate of the kappa statistic was considered almost perfect at 0.81–1.00, substantial at 0.61–0.80, moderate at 0.41–0.6, fair at 0.2–0.4, slight at 0–0.2, and poor at <0 [25,26].

The difference in days when comparing the reference US with the LMP or the Capurro method estimates was calculated (the LMP or the Capurro estimate against the US estimate, hereafter referred to as the GA difference); it was analyzed as a continuous variable and categorized into five groups: <−14; –14 to −8; –7 to +7; +8 to +14; and >+14 days. We chose those categories because they are cut-off points of discrepancy at which a clinician would replace an estimate of GA derived from the LMP with the US estimate: +/−7 days for first-trimester US and +/−14 days for second-trimester US [12]. Positive values indicate that the LMP- or Capurro-based GA estimate is earlier than the US-based estimate, and negative values indicate that the LMP- or Capurro-based GA estimate is later than the US-based estimate. Mean and SD values for the GA difference and the proportion of births within the categories of the GA difference were calculated within and compared across strata of maternal characteristics (for LMP) and infant characteristics (for the Capurro method). Analyses were performed using SPSS version 17.

## Results

Of the 1483 pregnant women studied, 1427 (97.4%) had information about the LMP; among those with this information, 1097 (76.9%) were certain about the date. Almost all the women (1401, 97.4%) had undergone at least one US examination during pregnancy and 1130 (78.6%) had undergone at least one US result at 7–20 weeks of gestation; however, only 256 (17.8%) had a US result from 10 weeks to 13 weeks 6 days of gestation. In all, 856 women (59.8%) had at least one US examination at 21–28 weeks of gestation, and 884 (61.7%) had at least one US examination at 29 or more weeks of gestation. The Capurro method was available for 1250 (87.5%) of the pregnancies (Figure 1).

**Figure 2:**
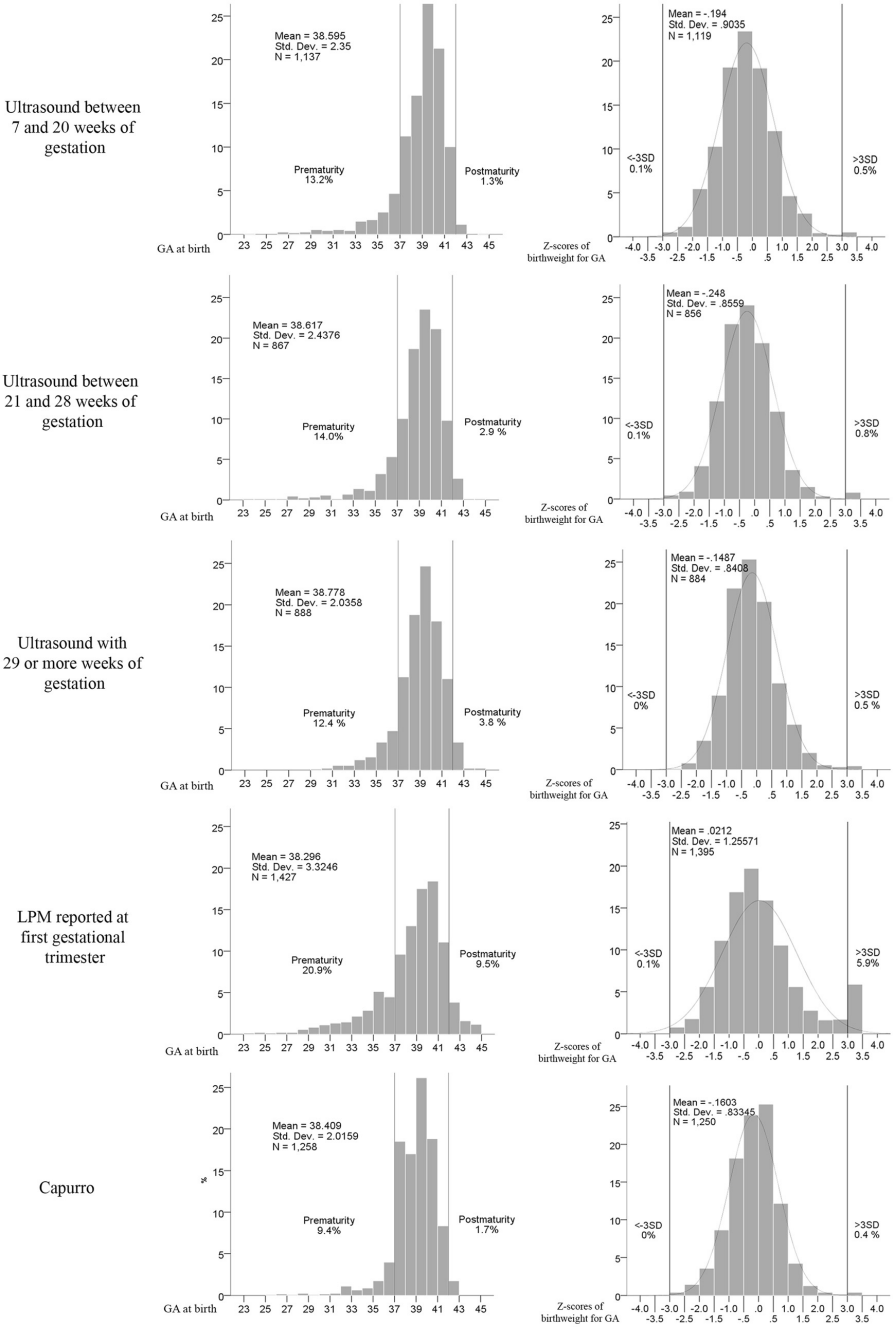
Gestational age (GA) at birth and Z-score of birthweight for GA distributions according to different methods of estimation.

The prematurity rate varied from 9.4% with the Capurro method to 20.9% with the LMP. For the reference US at 7 to 20 weeks of gestation, the rate was 13.2%; for US at 21–28 weeks and 29+ weeks, the rates were 14.0% and 12.4%, respectively (Figure 2).

In Table 1 the reference US result was compared with the other methods in two different ways: first, for all women that had undergone the two methods being compared; and second, excluding outlier values of Z scores for birth weight for GA (≤ −3 and ≥ +3 SD). For each method in the comparison, the sample in Table 1 is smaller than that in Figure 2 since the women had to have had an US examination at 7–20 weeks of gestation to be eligible for the comparison.

**Table 1:** **Distribution of prematurity and postmaturity rates along selected methods and comparison with 7**–**20**-**week ultrasound**-**based estimates of gestational age at birth**—**excluding outliers of birth weight for gestational age**

	**Reference 7–20-week ultrasound**		**21-28 weeks ultrasound**		**29+ weeks ultrasound**		**Capurro method**		**Last menstrual periodv (sure)**		**Last menstrual period (all)**		**Algorithm**
	**all**	**excluding outliers**	**all**	**excluding outliers**	**all**	**excluding outliers**	**all**	**excluding outliers**	**all**	**excluding outliers**	**all**	**excluding outliers**	**excluding outliers**
	**n (%)**	**n (%)**	**n (%)**	**n (%)**	**n (%)**	**n (%)**	**n (%)**	**n (%)**	**n (%)**	**n (%)**	**n (%)**	**n (%)**	**n (%)**
Prematurity	150 (13.2)	139 (12.5)	92 (14.8)	87 (14.2)	96 (13.6)	92 (13.2)	94 (9.8)	91 (9.6)	143 (16.9)	111 (13.8)	191 (17.7)	143 (14.0)	180 (12.5)
Term	972 (85.5)	959 (86.2)	513 (82.6)	510 (83.2)	585 (82.9)	583 (83.4)	850 (88.4)	843 (88.6)	625 (73.8)	614 (76.5)	780 (72.1)	768 (75.2)	1227 (85.3)
Post-maturity	15 (1.3)	14 (1.3)	16 (2.6)	16 (2.6)	25 (3.5)	24 (3.4)	17 (1.8)	17 (1.8)	79 (9.3)	78 (9.7)	111 (10.3)	110 (10.8)	31 (2.2)
Total	1137	1112	621	613	706	699	961	951	847	803	1082	1021	1438
Prematurity*													
Chi-square	-	-	0.77	0.85	0.06	0.11	5.86	4.44	5.28	0.719	8.52	1.05	0.27
P-value	-	-	NS	NS	NS	NS	<0.05	<0.05	<0.05	NS	<0.01	NS	NS
Kappa	-	-	0.76	0.75	0.63	0.63	0.63	0.65	0.55	0.60	0.52	0.56	-
Sensitivity	-	-	0.85	0.84	0.78	0.77	0.61	0.63	0.73	0.71	0.71	0.71	-
Specificity	-	-	0.96	0.96	0.94	0.94	0.97	0.97	0.91	0.94	0.90	0.94	-
PPV	-	-	0.74	0.74	0.59	0.60	0.74	0.77	0.52	0.61	0.50	0.61	-
PNV	-	-	0.98	0.98	0.97	0.97	0.95	0.95	0.96	0.96	0.96	0.96	-
**Post**-maturity**													
Chi-square	-	-	3.67	3.82	10.1	9.32	0.706	0.757	69.0	72.1	82.8	88.2	3.27
P-value	-	-	0.055	0.051	<0.01	<0.01	NS	NS	<0.01	<0.01	<0.01	<0.01	NS
Kappa	-	-	0.262	0.262	0.048	0.050	−0.016	−0.016	0.099	0.067	0.142	0.091	-
Sensitivity	-	-	0.98	0.98	0.97	0.97	0.98	0.98	0.91	0.91	0.90	0.90	-
Specificity	-	-	0.50	0.50	0.14	0.14	0.00	0.00	0.33	0.36	0.47	0.50	-
PPV	-	-	1.00	0.99	0.99	0.99	0.99	0.99	0.99	0.99	0.99	0.99	-
NPV	-	-	0.19	0.19	0.04	0.04	0.00	0.00	0.05	0.05	0.06	0.06	-

* prematurity vs. term and post-maturity combined.

** post-maturity vs. term and prematurity combined.

For prematurity, the kappa coefficient indicated a substantial or moderate agreement of all methods compared with the reference US. For postmaturity, the kappa coefficient indicated a fair agreement for 20–28 weeks US and a slight or poor agreement for the other methods (Table 1).

Regarding sensitivity and specificity measurements, US at 21–28 weeks had the highest sensitivity and the Capurro the highest specificity for evaluating prematurity. For postmaturity, US at 20–28 weeks and the Capurro method had a very high sensitivity (0.98). However, all methods had a very low specificity, with the highest value being for US at 21–28 weeks (0.50) (Table 1).

Considering the difference in days for the GA estimate at birth, we compared the agreement rate of the LMP and the reference US in terms of the mother’s characteristics. In general, the agreement rate (+/− 7 days) was 65%; the disagreement was greater when over 14 days (<−14 days or > + 14 days) (Table 2). Compared with women aged 20–34 years, there was a tendency for greater over- or underestimation of the GA among adolescents; among the oldest women, the tendency to underestimate the GP was the most frequent (*P=* 0.007). The women who reported being certain of the LMP showed a higher rate of agreement with the reference US (68.7%) than the ones who were not sure (*P* <0.001). For all other variables studied, we did not find any statistically significant differences (Table 2).

**Table 2:** **Difference between the LMP and 7**–**20**-**week ultrasound**-**based estimates of gestational age at birth according to maternal characteristics and differences between the Capurro method and 7**–**20**-**week ultrasound**-**based estimates according to infant characteristics**

		**GA difference (in days): LMP - US estimate**						
	***n***	**Mean of difference (SD)**	**< −14**	**(−14 to −8)**	**±7**	**(+8 to+14)**	**> +14**	***P-*value**
**Total**	1082	−0.01 (2.4)	9.1	7.0	65.1	8.1	10.6	-
**City**								
Petrópolis	630	−0.01 (2.4)	8.6	7.0	65.9	7.9	10.6	0.941
Queimados	452	−0.01 (2.3)	10.0	7.1	63.9	8.4	10.6	
**Age**								
10 - 19 years	238	0.08 (2.6)	10.5	5.9	59.2	10.9	13.4	0.007
20 - 34 years	751	0.06 (2.3)	8.0	6.5	67.8	7.6	10.1	
≥ 35	93	−0.76 (2.5)	15.1	14.0	58.1	5.4	7.5	
**Ethnicity**								
White	391	0.05 (2.4)	8.7	6.1	65.2	8.7	11.3	0.989
Mixed race	452	−0.02 (2.4)	9.3	7.7	64.8	7.5	10.6	
Black	239	−0.08 (2.2)	9.6	7.1	65.3	8.4	9.6	
**Years of schooling**								
≤ 4	135	−0.31 (2.6)	14.1	11.9	58.5	7.4	8.1	0.120
5 to 8	475	0.03 (2.5)	8.6	6.7	64.4	8.2	12.0	
≥ 9	472	0.04 (2.2)	8.3	5.9	67.6	8.3	10.0	
**Marital Status**								
Married	813	0.03 (2.4)	8.9	7.5	64.6	7.0	12.1	0.012
Not married	269	−0.12 (2.2)	13.7	5.6	66.5	11.5	6.3	
**Parity**								
First child	487	0.01 (2.3)	7.6	7.2	67.1	7.4	10.7	0.388
Second or third child	459	0.09 (2.3)	9.7	6.0	65.1	8.6	10.7	
Forth or more	133	−0.39 (2.9)	12.8	10.5	57.1	9.0	10.5	
**Certainty of LMP date**								
Sure	847	0.01 (2.2)	7.7	6.0	68.7	8.3	9.3	<0.001
Not sure	234	−0.08 (3.0)	14.1	10.7	52.1	7.7	15.4	
		**GA difference (in days): *Capurro- *US estimate**						
	***n***	**Mean of difference (SD)**	**< −14**	**(−14 to −8)**	**±7**	**(+8 to+14)**	**> +14**	***P***-**value**
**Infant birth weight**								
< 2500	81	0.20 (2.0)	3.7	12.3	58.0	12.3	13.6	<0.001
2500-3499	630	−0.37 (1.8)	12.9	15.4	58.7	7.1	5.9	
3500- 3999	199	−0.37 (1.4)	7.5	13.6	71.9	5.0	2.0	
> 4000	49	0.38 (1.6)	2.0	14.3	57.1	16.3	10.2	
**Gestational age at birth**								
<=36	114	1.19 (1.9)	0.9	4.4	57.0	14.0	23.7	<0.001
37	108	0.81 (1.4)	0.0	3.7	60.2	20.4	15.7	
38-39	420	−0.25 (1.4)	6.9	16.0	66.7	7.4	3.1	
40-41	304	−1.11 ( 1.3)	20.4	20.1	58.2	1.3	0.0	
>=42	14	−3.93 (3.3)	64.3	28.6	7.1	0.0	0.0	

Abbreviations: *GA*, gestational age; *LMP*, last menstrual period; *SD*, standard deviation; *US*, ultrasound.

We also compared the agreement rate between the Capurro method and the reference US in strata of birth weight and GA at birth. For the babies classified by the reference US as premature or with 37 weeks of GA, the Capurro method was more likely to overestimate the GA (37.7%); for postmature babies (≥ 42 weeks), the Capurro method was more likely to underestimate the GA (92.9%). The agreement rate (+/− 7 days) was also more frequent for babies weighing 3500–3999 g (71.9%) (Table 2).

Based on the comparisons shown in Tables 1 and 2, the entrance criteria in the algorithm of GA at birth estimation were as follows: US at 7–20 weeks (n= 1110, 77.2%); US at 21–28 weeks (n= 234, 16.3%); US at 29+ weeks (n=47, 3.3%); and LMP (n=47, 3.3%), with a total of 1438 newborns classified. Owing to the poor agreement rate between the reference US and the Capurro method for prematurity (even after excluding outliers of birth weight for GA; Table 1), the data were not used in the algorithm. Only 45 newborns (3%) were not classified, either because there was no information available for the GA or the available information was excluded as a result of being an outlier of birth weight for GA.

Finally, Table 3 shows that GA estimates at birth using the algorithm were very similar to those of the reference US. The prematurity rate estimate was of 12.5% with both methods.

**Table 3:** **Detailed comparison of 7**–**20**-**week ultrasound**-**based and algorithm**-**based estimates of gestational age at birth**—**excluding outliers of birth weight for gestational age**

	**Reference US (7–20 weeks)**			**Algorithm**		
**Weeks**	***n***	**%**	**Cum %**	***n***	**%**	**Cum %**
**22**-**29**	9	,8	,8	14	1,0	1,0
**30**	4	,4	1,2	4	,3	1,2
**31**	6	,5	1,7	6	,4	1,7
**32**	5	,4	2,2	6	,4	2,1
**33**	16	1,4	3,6	22	1,5	3,6
**34**	19	1,7	5,3	24	1,7	5,3
**35**	28	2,5	7,8	40	2,8	8,0
**36**	52	4,7	12,5	64	4,4	12,5
**PREMATURITY**	**139**	**12,5**		**180**	**12,5**	
**37**	128	11,5	24,0	164	11,4	23,9
**38**	179	16,2	40,2	235	16,3	40,2
**39**	302	27,1	67,3	388	27,0	67,2
**40**	238	21,4	88,7	290	20,2	87,4
**41**	111	10,0	98,7	149	10,4	97,8
**42**	13	1,2	99,8	27	1,9	99,7
**>=43**	2	,2	100,0	5	,3	100,0
**Total**	**1112**	100,0		**1438**	100,0	

Abbreviations: *US*, ultrasound.

## Discussion

This study found differences in GA estimates for all methods compared with 7–20 weeks US-based GA estimates (used as the reference). We were concerned about using birth weight distributions to minimize possible errors. Some studies have also used birth weight distributions when comparing different methods of GA estimation [27-29]. In our population, this method was very appropriate since we found considerable evidence of misclassification of GA based on LMP, with 6% outliers in the curve for birth weight for GA.

A limitation of this study is that only users of the public health care system were included in our sample, so we cannot generalize this finding for users of the private care sector. They would differ from the group studied in terms of socioeconomic and obstetric characteristics. Another limitation is that the US examinations used in the comparisons were not always performed by the same individual, and there was a difference in the personnel performing the US between the two cities. A classification bias in the US GA estimates could have been introduced because only in city 2 did a sonographer from our research team take measurements, and that individual would have been more dedicated to the research effort, producing less GA classification errors. Moreover, as the reference group, we used US examinations carried out at 14–20 weeks of gestation, though the ideal period is at 10–13 weeks. For these matters our results of sensitivity and specificity for prematurity may have been under or overestimated. In addition, this study was unable to compare postmaturity rates owing to the very low incidence (1.5%).

In the United States, LMP-based measures for GA consistently produce high false-positive rates for preterm and high false rates for post-term. The results in this study are consistent with those conducted in the United States [12,27]. However, the comparability with our population is limited owing to great differences in the socioeconomic conditions; for example, schooling and per capita income are much lower in Brazil than in the United States. Even in the United States, differences have been reported in the agreement rate according to socioeconomic level [12,27] and ethnicity [7,12,27]. In the current study, the discrepancy found between LMP and early US-based GA estimates could be explained in part by the low socioeconomic level of the women who enrolled. Indeed, despite this being a homogeneous socioeconomic group (users of the public health system), we identified more accentuated patterns of discordance for adolescents and with regard to marital status.

We found that the LMP overestimated the prematurity rate and the Capurro method underestimated it, though later US showed good agreement with the reference US (at 7–20 weeks). Curiously, we found that the LMP overestimated the prematurity rate in 58% of cases; this is different to what has been found in other studies, where the prematurity rate was underestimated by the LMP [9,27]. Another unusual finding was that the women in our study tended to produce errors of great magnitude: misclassification of over 2 weeks was more frequent than that for 1–2 weeks; this raises the possibility that these women made a mistake about the month of their LMP. Though the interviewers asked, “What was the first day of your last menstrual period?” the participants may have taken that to mean the first day when their LMP failed to occur, which would lead to an underestimation of the GA. We hypothesize that this misclassification of LMP is closely related to both the socioeconomic and reproductive characteristics of the study participants, for example, the lack of family planning and the inappropriate use of the contraceptive pill (the method most used in Brazil), which deregulates the menstrual cycle. A study in the city of Rio Grande, Rio Grande do Sul, Brazil, found that for the public health service users (in which most women belong to the low socioeconomic group) 65% of pregnancies were not planned [30].

Recent literature shows that later US results tend to overestimate the prematurity rate [31]. We found a slight, but not statistically significant, overestimation of the prematurity rate when we compared US at 21–28 weeks with the reference US (Table 2). Moreover, US at 29+ weeks did not overestimate the prematurity rate as had been expected. A possible explanation is that all premature babies under the age of 29 weeks were excluded when this method was taken into consideration, thereby reducing the prematurity rate to a level where it was similar to the reference US.

## Conclusions

This study underlines the care that should be taken when comparing the distribution of the GA in different localities when they are calculated using different methods. Another important aspect to consider is the individual risk of health care professionals in assessing the GA when the indication of interruption of pregnancy in this population leads to an iatrogenic prematurity. This situation is even more critical if there is no clinical indication of pregnancy—an event not uncommon in Brazil, as other studies have shown [32].

## Abbreviations

CE: Clinical estimates; LMP: Last menstrual period; NICE: National Institute for Health and Clinical Excellence; NPV: Negative predictive value; PPV: Positive predictive value; US: Ultrasound.

## Competing interests

The authors declare that they have no competing interests.

## Authors’ contributions

MCL designed the study; MCL and APEP conducted the research; APEP analyzed the data; MCL, APEP, MHB, MABD and SGNG wrote the paper; APEP had primary responsibility for the final content. All authors read and approved the final manuscript.
